# Retraction: Inhibition of Spontaneous Recovery of Fear by mGluR5 after Prolonged Extinction Training

**DOI:** 10.1371/journal.pone.0237058

**Published:** 2020-07-29

**Authors:** The PLOS ONE Editors

Following the publication of this article [1], concerns were raised regarding Figs 5 and 6:

- The bands in the left panel of Fig 5C appear similar to the bands in the right panel of Fig 5C. The authors state that there are differences between the backgrounds of the blots, indicating these are different blots, but no explanation has been provided regarding the level of similarity between the bands in the left and the right blot.
- The figure legend of Fig 5 states that S and I values were normalised to total protein for quantification, but the total protein control sample appears to be missing from the blots presented in Fig 5C.
- The PSD-95 panel and the Actin panel of Fig 6C look similar to the SAP-97 panel and the Actin panel of Fig 6D respectively. The authors indicated that the same panels from Fig 6C were used for Fig 6D inadvertently and have provided replacement panels for Fig 6D.

The individual level data for Fig 5A and 5B, as well as Fig 6A–6D have been provided by the authors, but the original uncropped blots underlying the blots presented in Figs 5A, 5C, 6B, 6C and 6D are no longer available.

In light of the concerns affecting multiple figure panels that question the integrity of these data, the *PLOS ONE* Editors retract this article.

CHC, OJM, and PWG agreed with the retraction. SCM, CCW, and MJO either did not respond directly or could not be reached.
